# Development of symptom-focused outcome measures for advanced and indolent systemic mastocytosis: the AdvSM-SAF and ISM-SAF^©^

**DOI:** 10.1186/s13023-021-02035-5

**Published:** 2021-10-09

**Authors:** Fiona Taylor, Cem Akin, Roger E. Lamoureux, Brad Padilla, Tanya Green, Anthony L. Boral, Iyar Mazar, Brenton Mar, Alan L. Shields, Frank Siebenhaar

**Affiliations:** 1 Adelphi Values, 225 Franklin St 10th Floor, Boston, MA 02110 USA; 2 University of Michigan, Ann Arbor, MI USA; 3 Blueprint Medicines, Cambridge, MA USA; 4 Dermatological Allergology, Department of Dermatology and Allergy, Charité - Universitätsmedizin Berlin, corporate member of Freie Universität Berlin, Humboldt-Universität zu Berlin, and Berlin Institute of Health, Berlin, Germany; 5 Present Address: Sanofi, Cambridge, MA USA

**Keywords:** Content validity, Instrument development, Patient-reported outcomes, Advanced systemic mastocytosis, Indolent systemic mastocytosis

## Abstract

**Background:**

Advanced systemic mastocytosis (AdvSM), indolent systemic mastocytosis (ISM), and smoldering systemic mastocytosis (SSM) are rare diseases characterized by neoplastic mast cell infiltration of more than one organ. A content-valid patient-reported outcome (PRO) questionnaire that assesses relevant signs and symptoms that are important and understandable to individuals with a condition is critical for assessing new treatment benefit as well as supporting product labeling claims. Notably, no such PRO questionnaire has been developed in accordance with regulatory and scientific guidelines for use in AdvSM, ISM, and SSM patient populations. To fill that gap, this study documents the development and content validity of instruments evaluating signs and symptoms of systemic mastocytosis.

**Methods:**

A review of peer-reviewed literature, advice meetings with clinical therapeutic area experts, patient concept elicitation interviews, concept selection and questionnaire construction meetings, and patient cognitive debriefing interviews were conducted, and regulatory feedback was incorporated.

**Results:**

For AdvSM, 26 sign- and symptom-level concepts were identified in literature, 39 by clinicians, and 33 by patients. For ISM/SSM, 38 sign- and symptom-level concepts were identified in the literature, 39 by clinicians, and 57 by patients. Two patient-reported instruments, the Advanced Systemic Mastocytosis Symptom Assessment Form (AdvSM-SAF) and Indolent Systemic Mastocytosis Symptom Assessment Form (ISM-SAF)(©Blueprint Medicines Corporation), were developed based on consolidated findings. Cognitive debriefing interviews with AdvSM and ISM patients showed the AdvSM-SAF and ISM-SAF were understood and interpreted as intended by the majority of patients.

**Conclusion:**

The AdvSM-SAF and ISM-SAF are content-valid tools measuring symptoms from AdvSM and ISM patients’ perspective.

**Supplementary Information:**

The online version contains supplementary material available at 10.1186/s13023-021-02035-5.

## Introduction

Systemic mastocytosis is a rare mast cell neoplasm of more than one organ driven by the KIT D816V mutation and is divided into different subclassifications [1], of which advanced systemic mastocytosis (AdvSM), indolent systemic mastocytosis (ISM), and smoldering systemic mastocytosis (SSM) are the primary types [2]. Specifically, AdvSM is a rare condition associated with shortened survival, comprising three subtypes: mast cell leukemia (MCL), aggressive systemic mastocytosis (ASM), and systemic mastocytosis with an associated hematologic (non-MC lineage) neoplasm (AHN) [3]. ISM often manifests with skin and other organ involvement with associated symptoms [3]. SSM is similar to ISM in its symptomatology, but has a worse prognosis, and was considered a subtype of ISM prior to the 2016 WHO reclassification of systemic mastocytosis [3]. In all three types of systemic mastocytosis, abnormal activation of mast cells leads to a significant, severely debilitating symptom burden [4].

A content-valid patient-reported outcome (PRO) questionnaire that assesses concepts relevant and important to individuals with a condition, in a way that is understandable to respondents, is important for assessing the efficacy of novel treatments in clinical studies, as well as supporting product labeling claims. The Memorial Symptom Assessment Scale (MSAS) [5] was developed for use in a wide variety of cancers; however, despite being used in AdvSM trials [6], no AdvSM patients were involved in its development. For ISM, the Mastocytosis Quality of Life Questionnaire (MC-QoL) was developed with a focus on evaluating the impact of ISM on patients’ lives rather than on symptom severity [7], and implemented a two-week recall period; measuring symptom severity over a shorter recall period may be more defensible for the evaluation of treatment benefit in a clinical trial setting. The Mastocytosis Activity Score (MAS) was developed as a prospective instrument to assess symptom severity in ISM patients [8]; however, while relying on European standards it is not considered to be consistent with FDA regulatory guidelines. Notably, no PRO instrument has been developed in such a way for use in AdvSM and ISM patient populations [9–12].

In order to address the lack of appropriate PRO instruments to assess symptom data, particularly in AdvSM, a literature review, expert advice meetings (EAMs), concept elicitation (CE) interviews, questionnaire construction procedures, and cognitive debriefing (CD) interviews were conducted to inform the development of new PRO instruments. To determine whether a single PRO instrument that is consistent with regulatory expectations could be developed for both AdvSM and ISM, CE interviews and EAMs were conducted for both conditions. This study documents the development and content validity of the AdvSM Symptom Assessment Form (AdvSM-SAF) and ISM Symptom Assessment Form (ISM-SAF), supporting the future use of these instruments in evaluating the signs and symptoms of systemic mastocytosis.

## Methods

The qualitative research supporting AdvSM and ISM questionnaire development was completed in five stages: (1) a review of the peer-reviewed literature on systemic mastocytosis; (2) EAMs with clinical experts (jointly for AdvSM and ISM); (3) CE interviews (jointly for AdvSM and ISM); (4) questionnaire construction (independently for AdvSM and ISM); and (5) CD interviews (independently for AdvSM and ISM). For both the AdvSM and ISM tools, regulatory feedback was sought and incorporated at key points during the development process to ensure alignment with best practices for PRO development and measurement.

### Targeted literature review

**Fig. 1:**
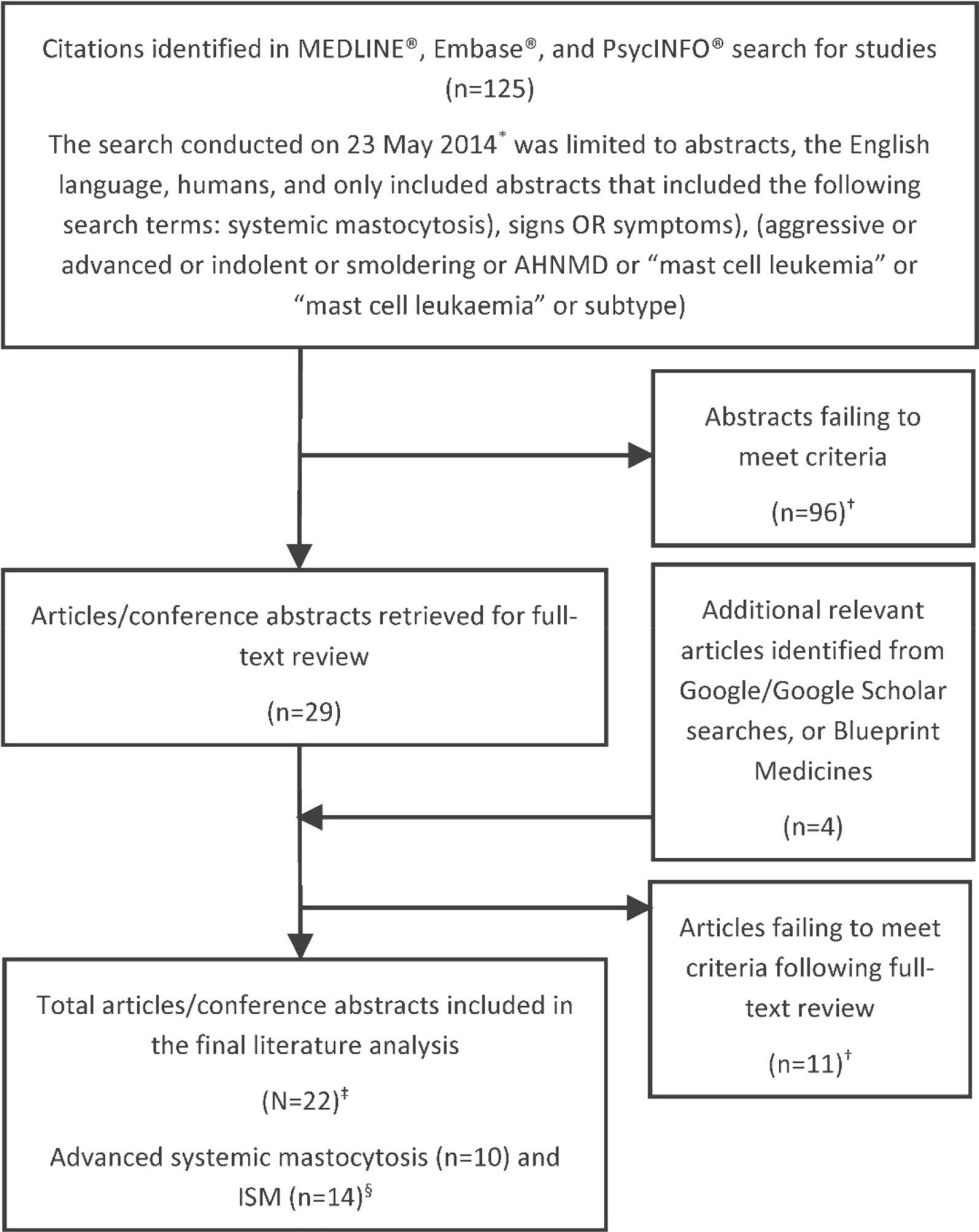
Literature search flow diagram. *Databases: Ovid MEDLINE(R) 1946 to Present with Daily Update; Embase 1988 to 2014 Week 20; PsycINFO 1967 to 2014 week 3; Date of search: 23 May 2014. ^†^Did not meet inclusion criteria. ^‡^Total articles/conference abstracts reviewed (n = 33). ^§^Not mutually exclusive; two articles discussed both AdvSM and ISM symptoms

A search strategy was developed to identify peer-reviewed literature published in English, focusing on the signs and symptoms of systemic mastocytosis subtypes of interest (AdvSM, ISM, and SSM) in humans (for details, see Fig. 1, as well as Additional file 1: Table S1). The search strategy was executed in the OvidSP platform (supplemented by Google/Google Scholar search); subsequently, abstracts were exported and reviewed using Abstrackr (abstrackr.cebm.brown.edu, a web-based screening tool), and relevant articles were selected for full-text review. Patient-reportable signs and symptoms for AdvSM and ISM were catalogued into data extraction tables. Separate literature-focused conceptual models (CMs) of AdvSM and ISM were developed, outlining the disease process and signs and symptoms of each condition. Separate concept description tables were developed to describe the signs and symptoms, as reported in the literature, for AdvSM and ISM.

### Expert advice meetings (i.e., interviews with clinical therapeutic area experts)

Five clinical therapeutic area experts in AdvSM and/or ISM were interviewed, using a semi-structured interview guide developed to elicit spontaneous responses. At the conclusion of the EAMs, experts were asked to review the systemic mastocytosis sign and symptom CMs derived from the previously conducted literature review. Information was collected in order to characterize the experts (e.g., years of experience treating patients with systemic mastocytosis, medical specialty, approximate number of systemic mastocytosis patients seen in a typical year, typical work setting).

The EAMs, which lasted approximately 60 min each, were conducted one-on-one via telephone, audio-recorded, transcribed, and anonymized. Transcripts were coded in ATLAS.ti (Scientific Software Development GmbH, Berlin, Germany) to organize and catalog descriptions of the systemic mastocytosis sign and symptom concepts that experts reported. Coded data were analyzed to determine concept frequency (i.e., the number of experts who reported each unique concept) and to arrive at characterizations (i.e., qualitative descriptions) for each unique concept. Separate expert-based CMs were developed for the sign- and symptom-level concepts related to AdvSM and ISM based on the results of the meetings with experts.

### Concept elicitation interviews

#### Study overview

Participants were recruited through an advocacy group or clinical sites in the US and Germany with study materials approved by a centralized independent review board (IRB), Copernicus Group IRB, and, for clinical-site interviews, through local ethics committees associated with Stanford University in the US and Mannheim University in Germany. Participants were consented and considered eligible to participate if they were adults with a documented, clinician-confirmed diagnosis of AdvSM, ISM, or SSM. Recruitment targets of 15 patients with AdvSM and 15 patients with ISM or SSM were specified.

#### Interview conduct

One-on-one patient interviews were conducted in person or via telephone and lasted approximately 60 min each. Trained interviewers followed a semi-structured CE interview guide designed to elicit spontaneous responses from patients regarding their experience with AdvSM, ISM, or SSM. Participant characteristics were collected through a demographic and health information form (DHIF).

#### Qualitative data management and coding

All interviews were audio-recorded and transcribed (of note, German language audio-recordings were transcribed in German and subsequently translated into US English), and transcripts were coded in ATLAS.ti to organize and catalog patient descriptions of the AdvSM, ISM, or SSM sign and symptom concepts participants reported. Coded data were analyzed by condition to determine concept frequency (i.e., the number of AdvSM, ISM, or SSM patients who reported each concept), as well as concept clarification (i.e., patient language used to describe each systemic mastocytosis sign or symptom with respect to its salient aspects [e.g., severity, frequency, duration]). Analyses of saturation, which characterizes the point at which no new or relevant information is likely to be gained from conducting additional interviews and serves as evidence of the adequacy of the study’s sample size, were performed [10, 13]. Patient-based CMs that included sign and symptom concepts relevant to the condition were developed for AdvSM and ISM, separately, based on the results of the CE interviews. Descriptive data from case report forms or screening documents and DHIFs were aggregated and presented in tables in order to summarize the demographic and health characteristics of the CE interview study samples.

### Concept selection and questionnaire construction for the Advanced Systemic Mastocytosis Symptom Assessment Form (AdvSM-SAF) and Indolent Systemic Mastocytosis Symptom Assessment Form (ISM-SAF)

Prior to questionnaire development meetings, the following materials were distributed to the instrument developers: (a) findings tables containing relevant CE interview quotes from AdvSM or ISM patients and (b) a concept tracking matrix (CTM) highlighting concepts reported by AdvSM or ISM patients during the CE interviews, by clinical therapeutic area experts during EAMs, and/or in the peer-reviewed literature. To draft the questionnaires, meetings with measurement and clinical experts were held for AdvSM first and ISM subsequently, during which the CTM and the results of the CE interviews with AdvSM or ISM patients were leveraged to select symptom concepts that were the most relevant to the patient experience, to be targeted for measurement by the PRO questionnaire. Following selection of concepts, questionnaire instructions, items, and response choices were drafted (of note, structure and format of ISM items emulated patterns and stems of the previously drafted AdvSM items). Based on the questionnaire structure, conceptual frameworks were developed for each questionnaire, as well as developer definitions for each of the items to be included in the draft PRO questionnaires. Translatability assessments were performed for each questionnaire, and the questionnaires were designed and formatted for ePRO administration.

### Cognitive debriefing interviews for the AdvSM-SAF and ISM-SAF

#### Study overview

The CD interview study was conducted using the same recruitment procedures and eligibility criteria as described for the CE study and under the same IRB and ethics approvals. For AdvSM and ISM, 15 interviews were targeted for each subtype.

#### Interview conduct

One-on-one patient interviews were conducted by trained researchers either in person or via telephone and lasted approximately 60 min. Trained interviewers followed a semi-structured CD interview guide to debrief the draft questionnaires, the AdvSM-SAF and ISM-SAF. The interview guide was designed to collect data regarding participants’ understanding of each component of the AdvSM-SAF or ISM-SAF.

#### Data management and analysis

All interviews were audio-recorded and transcribed (of note, German language audio-recordings for AdvSM were transcribed in German and subsequently translated into US English), and transcript text was coded in ATLAS.ti to organize and catalog patient feedback on the AdvSM-SAF or ISM-SAF and responses to open-ended interview questions. Coded data were analyzed to determine participants’ ability to understand the questionnaire as intended, whether the targeted concepts mapped to the participants’ experience with AdvSM or ISM, and whether participants could select response options that reflected their own health status. Descriptive data from case report forms or screening documents and DHIFs were aggregated as described previously.

## Results

### Literature review

The search strategy was executed in OvidSP on 23 May 2014, returning 125 unique abstracts (for details, see Fig. 1 and Additional file 1: Table S1). Following initial review of the abstracts, 29 articles were selected for full-text review, and a total of 22 were included in the final analysis (Fig. 1).

### Expert advice meetings (interviews with clinical therapeutic area experts)

Five clinical therapeutic area experts from the US (n = 4) and United Kingdom (n = 1) were interviewed between July and August 2014. Three clinicians were interviewed regarding the cardinal signs and symptoms of both AdvSM and ISM, and one clinician each was interviewed regarding the cardinal signs and symptoms of AdvSM or ISM only. All clinicians had at least nine years of experience treating patients with systemic mastocytosis and treated at least 30 systemic mastocytosis patients per year. Two clinicians (40%) specialized in allergy and immunology, while one clinician each (20%) specialized in oncology, myeloproliferative disorders, and hematology (for details, see Additional file 1: Table S2).

### Advanced systemic mastocytosis

#### Literature review

**Fig. 2:**
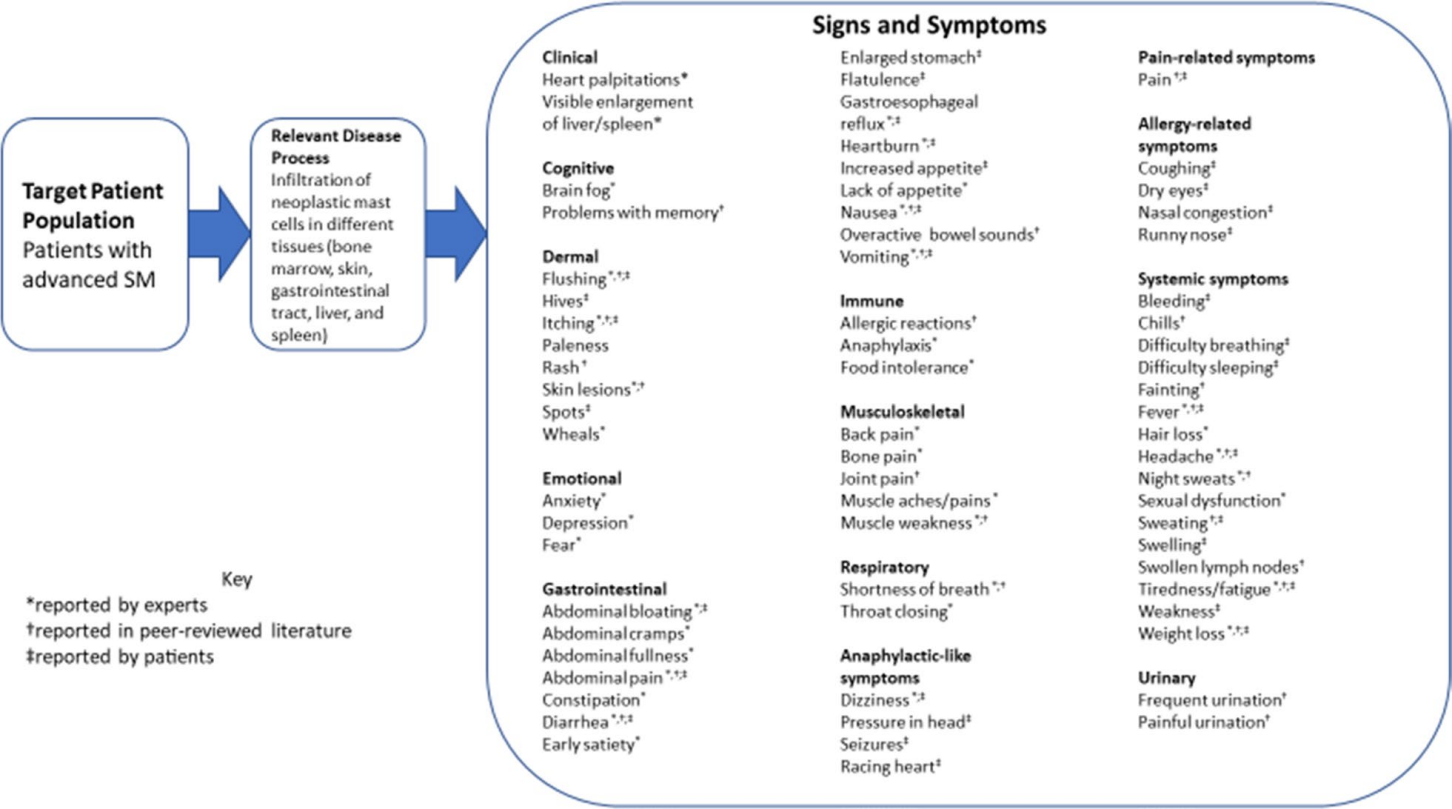
Harmonized literature, expert, and patient concept elicitation conceptual model for advanced systemic mastocytosis

A total of 26 sign- and symptom-level concepts were identified in the literature, presented in the harmonized conceptual model (CM) for AdvSM (Fig. 2).

#### Expert advice meetings with AdvSM clinical therapeutic area experts

Clinicians reported a total of 39 relevant sign and symptom concepts for AdvSM (harmonized CM for AdvSM, Fig. 2). All clinicians (n = 4, 100%) reported bone pain, diarrhea, itching, and weight loss; three clinicians (75%) reported abdominal bloating, abdominal pain, nausea, vomiting, lack of appetite, flushing, skin lesions, brain fog, shortness of breath, anaphylaxis, and fatigue.

#### Concept elicitation interviews

Fifteen participants with AdvSM were interviewed in the US (n = 8) and Germany (n = 7), and ultimately 12 participants were included in the analysis (three US patients were removed from the analysis group based on evaluation of diagnosis documentation). Of these participants, six (50%) were diagnosed with AHN and three each (25%) with ASM and MCL (demographic and health information, Table 1).

**Table 1:** AdvSM and ISM demographic and health characteristics: concept elicitation and cognitive briefing interviews

	AdvSM patients		ISM patients	
	CE interviews Total (N = 12) n (%)^*^	CD interviews Total (N = 13) n (%)^*^	CE interviews Total (N = 15)^†^ n (%)^*^	CD interviews Total (N = 10) n (%)^*^
*Age (years)*				
Range	38.9–77.5	49.8–82.6	26.7–69.0	29.4–56.7
Mean (standard deviation [SD])	62.1 (12.9)	68.7 (10.9)	48.3 (14.2)	45.0 (10.0)
*Gender*				
Female	7 (58.3%)	7 (53.8%)	14 (93.3%)	8 (80.0%)
Male	5 (41.7%)	6 (46.2%)	1 (6.7%)	2 (20.0%)
*Race*				
White	7 (58.3%)	12 (92.3%)	14 (93.3%)	10 (100.0%)
Not answered	4 (33.3%)	0 (0.0%)	–	–
Asian	1 (8.3%)	0 (0.0%)	–	–
American Indian or Alaska Native	0 (0.0%)	1 (7.7%)	1 (6.7%)	0 (0.0%)
*Ethnicity*				
Data not collected in Germany	7 (58.3%)	7 (53.8%)	–	–
No, not Spanish/Hispanic/Latino	4 (33.3%)	5 (38.5%)	13 (86.7%)	10 (100.0%)
Yes, Puerto Rican	–	–	1 (6.7%)	0 (0.0%)
Not answered	1 (8.3%)	1 (7.7%)	1 (6.7%)	0 (0.0%)
*Highest level of education*				
High school diploma (or GED) or less	3 (25.0%)	1 (7.7%)	2 (13.3%)	2 (20.0%)
Some college or certificate program	4 (33.3%)	7 (53.8%)	3 (20.0%)	2 (20.0%)
College or university degree (two- or four-year)	2 (16.7%)	4 (30.8%)	8 (53.3%)	3 (30.0%)
Graduate degree	3 (25.0%)	1 (7.7%)	1 (6.7%)	3 (30.0%)
Other	–	–	1 (6.7%)	0 (0.0%)
*Work status*				
Retired	6 (50.0%)	9 (69.2%)	1 (6.7%)	0 (0.0%)
On disability	3 (25.0%)	2 (15.4%)	7 (46.7%)	3 (30.0%)
Working part-time	3 (25.0%)	2 (15.4%)	3 (20.0%)	2 (20.0%)
Working full-time	1 (8.3%)	1 (7.7%)	3 (20.0%)	4 (40.0%)
Homemaker	–	–	2 (13.3%)	0 (0.0%)
Student	–	–	1 (6.7%)	0 (0.0%)
Unemployed	–	–	0 (0.0%)	1 (10.0%)
*Type of systemic mastocytosis*				
AHN	6 (50.0%)	8 (61.5%)	–	–
ASM	3 (25.0%)	2 (15.4%)	–	–
MCL	3 (25.0%)	3 (23.1%)	–	–
ISM	–	–	13 (81.3%)	10 (100.0%)
SSM	–	–	3 (18.7%)	0 (0.0%)
*Time since diagnosis (years)*				
Range	0.2–3.8	0.1–8.4	0.3–18.1	–
Mean (SD)	0.7 (1.0)	0.7 (2.6)	4.3 (4.1)	–
*Other health conditions (participant-reported)*^*‡*^				
Heart disease	2 (16.7%)	0 (0.0%)	1 (6.7%)	0 (0.0%)
High blood pressure	2 (16.7%)	4 (30.8%)	2 (13.3%)	2 (20.0%)
Chronic obstructive pulmonary disease (COPD)	0 (0.0%)	3 (23.1%)	0 (0.0%)	1 (10.0%)
High cholesterol	2 (16.7%)	0 (0.0%)	2 (13.3%)	0 (0.0%)
Asthma	–	–	2 (13.3%)	1 (10.0%)
Fibromyalgia	–	–	2 (13.3%)	2 (20.0%)
Liver disease	2 (16.7%)	1 (7.7%)	1 (6.7%)	0 (0.0%)
Kidney disorder	–	–	1 (6.7%)	0 (0.0%)
Thyroid disease	2 (16.7%)	2 (15.4%)	3 (20.0%)	1 (10.0%)
Depression/anxiety	1 (8.3%)	2 (15.4%)	8 (53.3%)	7 (70.0%)
Migraine headaches	–	–	4 (26.7%)	0 (0.0%)
Cancer	1 (8.3%)	1 (7.7%)	0 (0.0%)	2 (20.0%)
Stomach/intestinal disorder	1 (8.3%)	1 (7.7%)	3 (20.0%)	4 (40.0%)
None	2 (16.7%)	2 (15.4%)	3 (20.0%)	0 (0.0%)
Other	2 (16.7%)	3 (23.1%)	5 (33.3%)	3 (30.0%)
Not answered	1 (8.3%)	1 (7.7%)	0 (0.0%)	1 (10.0%)
*Treatments (current and past)*^*‡;§*^				
Tyrosine kinase inhibitors	Current: 6 (50.0%) Past: 3 (25.0%)	Current: 6 (46.2%) Historical: 3 (23.1%)	Current: 3 (18.8%) Past: 1 (6.3%)	Current: 1 (10.0%)
H1 antagonists	Current: 5 (41.7%) Past: 2 (16.7%)	Current: 4 (30.8%) Historical: 3 (23.1%)	Current: 16 (100.0%) Past: 3 (18.8%)	Current: 7 (70.0%) Past: 2 (20.0%)
Corticosteroids	Current: 5 (41.7%) Past: 1 (8.3%)	Current: 3 (23.1%) Historical: 2 (15.4%)	Current: 2 (12.5%) Past: 4 (25.0%)	Current: 1 (10.0%) Past: 6 (60.0%)
Proton pump inhibitor	Current: 5 (41.7%)	Current: 5 (38.5%)	Current: 3 (18.8%) Past: 1 (6.3%)	Current: 2 (20.0%) Past: 1 (10.0%)
H2 antagonists	Current: 4 (33.3%) Past: 1 (8.3%)	Current: 5 (38.5%) Historical: 1 (7.7%)	Current: 14 (87.5%) Past: 0 (0.0%)	Current: 6 (60.0%) Past: 2 (20.0%)
Non-steroidal anti-inflammatory drugs	Current: 4 (33.3%)	Current: 4 (30.8%)	Current: 3 (18.8%) Past: 0 (0.0%)	Current: 3 (30.0%) Past: 1 (10.0%)
Cytokine/immunomodulatory drugs	Current: 2 (16.7%) Past: 1 (8.3%)	Current: 1 (7.7%) Historical: 1 (7.7%)	Current: 2 (12.5%) Past: 3 (18.8%)	Past: 2 (20.0%)
Beta-adrenergic agonists	Current: 1 (8.3%)	–	Current: 3 (18.8%) Past: 0 (0.0%)	Current: 3 (30.0%) Past: 1 (10.0%)
Anti-IgE	–	–	Current: 0 (0.0%) Past: 1 (6.3%)	–
Cannabis	Current: 1 (8.3%)	–	–	–
Aldactone®	Current: 1 (8.3%)	–	–	–
Zofran®	Current: 1 (8.3%)	–	–	–
Tramadol	Current: 1 (8.3%)	–	–	–
Zaditor®	Current: 1 (8.3%)	–	–	–
Hydroxycarbamide	Current: 1 (8.3%)	–	–	–
Zoledronate	Current: 1 (8.3%)	–	–	–
Purine nucleoside analogues	Current: 0 (0.0%) Past: 2 (16.7%)	Historical: 3 (23.1%)	Current: 0 (0.0%) Past: 1 (6.3%)	–
Leukotriene antagonist	–	Current: 1 (7.7%)	Current: 7 (43.8%) Past: 0 (0.0%)	Current: 3 (30.0%)
Other	–	Current: 6 (46.2%)	Current: 2 (12.5%) Past: 4 (25.0%)	Current: 8 (80.0%) Past: 2 (20.0%)

*Unless otherwise noted

^†^Demographic data were not collected for one participant

^‡^Not mutually exclusive

^§^Counts are not provided for past treatments if these were not reported by any respondents and/or clinicians

Participants reported a total of 33 disease-related signs and symptoms across six domains: gastrointestinal symptoms, anaphylactic-like episodes, pain-related symptoms, dermal symptoms, allergy-related symptoms, and systemic symptoms (harmonized CM for AdvSM, Fig. 2). Saturation of concept was demonstrated; of the 33 concepts elicited during participant interviews, 29 concepts (87.9%) were elicited prior to the fourth quartile of interviews. The four concepts that emerged during the final quartile of interviews (acid reflux, dizziness, runny nose, and difficulty sleeping) could be considered to be idiosyncratic to the participant reporting them (considering each concept was reported by only a single participant). The most frequently reported concepts included vomiting (n = 9, 75.0%); abdominal pain and spots on the skin (n = 8 each, 66.7%); diarrhea, nausea, and tiredness/fatigue (n = 7 each, 58.3%); itching (n = 5, 41.7%); and flushing and weight loss (n = 4 each, 33.3%).

#### Questionnaire construction

An in-person concept selection and item generation meeting was held on 25 August 2015. Based on qualitative data generated from the CE interviews, a recall period of 24 h was selected for all draft items, since a daily diary was more likely to reflect the variability of symptom severity and allow patients to more accurately recall the severity of their symptoms. The draft 10-item questionnaire included the concepts of abdominal pain, nausea, vomiting, diarrhea, spots, itching, flushing, tiredness/fatigue, vomiting frequency, and diarrhea frequency. All items except for those relating to frequency (e.g., vomiting frequency, diarrhea frequency) assessed severity using an 11-point numeric rating scale (NRS).

#### Cognitive debriefing of the advanced systemic mastocytosis symptom assessment form (AdvSM-SAF)

A total of 13 patients (n = 6 US, n = 7 Germany) participated in the CD interviews. Of these, eight participants were diagnosed with AHN (61.5%), three with MCL (23.1%), and two with ASM (15.4%) (demographic and health information, Table 1).

Overall, participants demonstrated the ability to understand and interpret the AdvSM-SAF instructions (n = 11/12, 91.7% of instructions part one, n = 8/9, 88.9% of part 2), items (n ≥ 11, ≥ 91.7% of participants providing interpretable responses), and response options (≥ 77.8% of interpretable responses) as intended, and reported that the concepts assessed in the questionnaire were relevant to their experience of AdvSM. Five participants in Germany (45.5%) provided suggestions regarding the phrasing of the German translation of “flushing” for Item 5; five participants (41.7%) suggested either deleting or rewording the term “tiredness” from Item 6 (fatigue), which was also problematic when translated into German. A small number of participants either misinterpreted the response options for Item 8 (vomiting frequency) (n = 2, 18.2%) or 10 (diarrhea frequency) (n = 1, 16.7%), or suggested revising the ordering/formatting of those items (n = 2, 15.4%). All participants who completed in-person CD interviews found the AdvSM-SAF easy to complete using an electronic device.

Based on the results of the CD interviews, the word “tiredness” was removed from Item 6 (fatigue) in order to facilitate interpretation of the AdvSM-SAF in German, as well as to facilitate the translation of the questionnaire into other languages. Items 7 and 8 (vomiting severity and frequency), as well as Items 9 and 10 (diarrhea severity and frequency), were reversed so that the frequency item of the pair is presented to respondents first (AdvSM-SAF conceptual framework, Table 2).

**Table 2:** Conceptual framework for the AdvSM-SAF

Concept	Domain	General concept
Abdominal pain	Gastrointestinal symptom severity	Total symptom severity
Nausea		
Vomiting		
Diarrhea		
Spots	Skin symptom severity	
Itching		
Flushing		
Fatigue	Fatigue severity	
Vomiting frequency	Vomiting frequency	Vomiting frequency
Diarrhea frequency	Diarrhea frequency	Diarrhea frequency

### Indolent systemic mastocytosis and smoldering systemic mastocytosis

#### Literature review

**Fig. 3:**
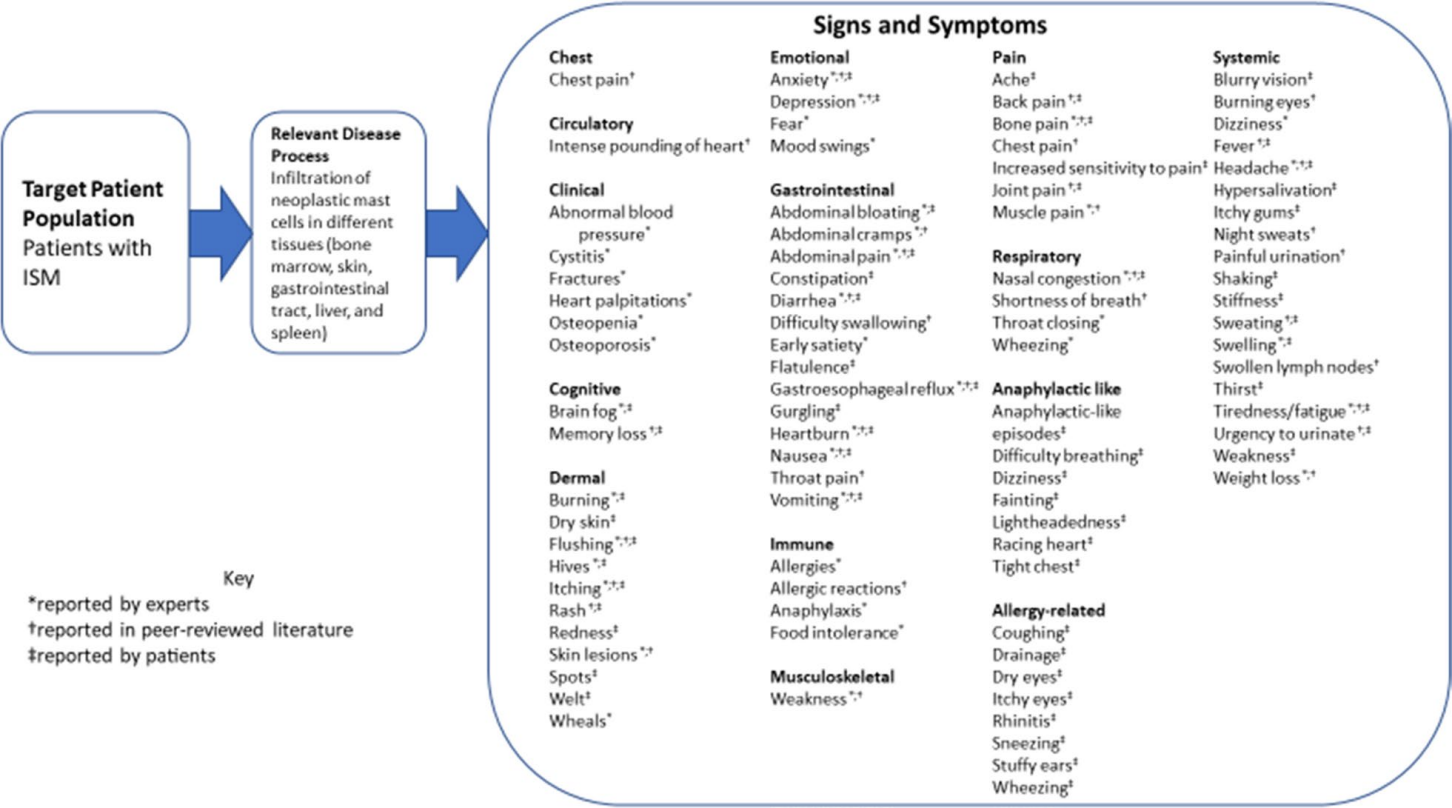
Harmonized literature, expert, and patient concept elicitation conceptual model for indolent systemic mastocytosis

A total of 38 sign- and symptom-level concepts were identified in the literature, presented in the harmonized CM for ISM (Fig. 3).

#### Expert advice meetings with ISM and SSM clinical therapeutic area experts

Clinicians reported a total of 39 relevant sign and symptom concepts for ISM and SSM (harmonized CM for ISM, Fig. 3). Overall, the most common symptoms, reported by all experts (n = 4, 100%), included anaphylaxis, brain fog, diarrhea, flushing, and itching. The next most commonly reported symptoms were abdominal bloating, fatigue, nausea, and skin lesions, which were reported by three experts (75%) each.

#### Concept elicitation interviews

Sixteen participants with ISM (n = 13, 81.3%) or SSM (n = 3, 18.7%) participated in the CE interviews, all of whom were recruited via The Mastocytosis Society (TMS) in the US (demographic and health information, Table 1).

Participants reported a total of 57 sign and symptom concepts across eight domains: gastrointestinal symptoms, anaphylactic-like episodes, pain-related symptoms, dermal symptoms, allergy-related symptoms, emotional symptoms, cognitive symptoms, and systemic symptoms (harmonized CM for ISM, Fig. 3). Saturation was considered reached; of the 57 sign and symptom concepts that were elicited spontaneously from participants, 44 concepts (77.2%) were elicited in the first 75% of interviews. Study investigators evaluated the 13 sign and symptom concepts elicited in the final quartile of interviews, and the results suggested that the sample size for the study was adequate. The most frequently reported concepts included spots, itching, and diarrhea (n = 12 each, 75.0%); tiredness/fatigue and flushing (n = 11 each, 68.8%); abdominal pain, bone pain, and cognitive issues/brain fog (n = 10 each, 62.5%); and nausea (n = 8, 50.0%).

#### Questionnaire construction

Three teleconference meetings were held between March 2017 and April 2017. Based on concepts that were relevant and important to patients with ISM as reported in the CE interviews that were not relevant and important to patients with AdvSM, the development team determined that a separate PRO instrument for ISM was needed. A recall period of 24 h was selected for all draft items, since a daily diary was more likely to accurately reflect the variability of symptom severity and allow patients to more accurately recall the severity of their symptoms. The draft 13-item questionnaire included the concepts of abdominal pain, nausea, diarrhea, spots, itching, flushing, bone pain, joint pain, fatigue, dizziness, brain fog and an alternate question on mental confusion (based on interpretation and translatability concerns), headache, and diarrhea frequency. All items except for the one relating to frequency assessed severity using an 11-point NRS.

#### Cognitive debriefing of the Indolent Systemic Mastocytosis Symptom Assessment Form (ISM-SAF)

Ten participants with ISM participated via telephone in the CD interviews, all of whom were recruited via TMS in the US (demographic and health information, Table 1). Overall, participants demonstrated the ability to understand and interpret the ISM-SAF instructions (n = 10/10, 100.0%), items (n ≥ 8/10, ≥ 80.0%), and response options (all participants providing an interpretable response, 100.0%) as intended, and reported that the concepts assessed in the questionnaire were relevant to their experience of ISM. Participants were able to distinguish between Item 1 (bone pain) and Item 2 (joint pain) without any issues.

While all participants (n = 10, 100.0%) interpreted Item 10 (brain fog) as intended by developers, fewer participants (n = 8; 80.0%) interpreted Item 10a (mental confusion), which was originally included as an alternative to Item 10 (brain fog) following a translation feasibility assessment, as intended. Therefore, the item assessing “mental confusion” was recommended for deletion. Separate from the modifications made in response to CD interview feedback, the joint pain item of the ISM-SAF (Item 2) was removed from the questionnaire due to regulatory feedback, the lack of relevance to the condition, and redundancy with the symptom of bone pain (ISM-SAF conceptual framework, Table 3).

**Table 3:** Conceptual framework for the ISM-SAF

Concept	Domain	General concept
Abdominal pain	Gastrointestinal symptom severity	Total symptom severity
Nausea		
Diarrhea		
Spots	Skin symptom severity	
Itching		
Flushing		
Bone pain	Bone pain	
Fatigue	Fatigue	
Dizziness	Dizziness	
Brain fog	Brain fog	
Headache	Headache	
Diarrhea frequency	Diarrhea frequency	Diarrhea frequency

## Discussion

Based on the results of the literature review, EAMs with clinical experts, and interviews with patients, the AdvSM-SAF and ISM-SAF were developed to measure relevant and important symptoms of AdvSM and ISM from the patient perspective. Cognitive debriefing of both instruments with patients with the respective conditions demonstrated the content of each questionnaire to be relevant, the items easy to comprehend, and the response options clear and appropriate. Together, results presented here support the conclusion that both the AdvSM-SAF and ISM-SAF are content valid in the respective target patient populations.

Although the sample size in the AdvSM CE interviews was small, in part because rigorous verification of diagnoses resulted in exclusion of some participants and in part due to the rarity of the disease, saturation was demonstrated with the sample of 12 participants, as shown by the decrease in emergence of novel concepts [14]. In the ISM CE interviews, the sample size was in line with recommendations for eliciting data to inform PRO instrument development; prior research has demonstrated that more than 84% of symptom concepts emerge by the 10th interview [15] and, in rare disease, 92% by the 12th interview [16]. The alignment between concepts reported in the literature and expert interviews further supports the comprehensiveness of the concepts emerging from the CE patient interviews. Similarly, the responses from participants in the CD interviews for both the AdvSM-SAF and ISM-SAF were largely consistent, indicating the sample size was adequate.

Throughout the development of the AdvSM-SAF and ISM-SAF, the development team sought regulatory feedback to ensure the instruments aligned with regulatory expectations for instruments intended for use in clinical trials and that the instruments were fit for the intended purpose. This led to the removal of the joint pain item from the ISM-SAF.

## Conclusion

The content validity of the AdvSM-SAF and ISM-SAF was demonstrated as part of this research, which is critical to establish prior to considering evidence related to the psychometric performance of an instrument [9]. Accordingly, the AdvSM-SAF and ISM-SAF will be implemented in future studies to evaluate the psychometric performance of their scores when administered to patients with AdvSM and ISM. Additionally, quantitative data will be used to inform guidelines as to the clinical meaning and interpretation of observed between-group differences and within-person change. The content validity evidence presented here, along with the psychometric and score interpretation information to be collected, can be used to demonstrate that the instruments are fit-for-purpose for evaluating the clinical benefit of treatment interventions in systemic mastocytosis.

## Supplementary Information

**Additional file 1: Table S1.** Literature search strategy executed on 23 May 2014. **Table S2.** Description of experts.

## Data Availability

The datasets used and/or analyzed during the current study are available from the corresponding author on reasonable request.

## Abbreviations

AHN: Systemic mastocytosis with an associated hematologic (non-MC lineage) neoplasm
ASM: Advanced systemic mastocytosis
AdvSM-SAF: Advanced Systemic Mastocytosis Symptom Assessment Form
CD: Cognitive debriefing
CE: Concept elicitation
CM: Conceptual model
CTM: Concept tracking matrix
DHIF: Demographic and health information form
EAM: Expert advice meetings
IRB: Independent review board
ISM: Indolent systemic mastocytosis
ISM-SAF: Indolent Systemic Mastocytosis Symptom Assessment Form
MCL: Mast cell leukemia
PRO: Patient-reported outcome
SSM: Smoldering systemic mastocytosis
