# Supplementary material for: Development of symptom-focused outcome measures for advanced and indolent systemic mastocytosis: the AdvSM-SAF and ISM-SAF©
Source: Orphanet J Rare Dis. 2021 Oct 9;16:414. doi: 10.1186/s13023-021-02035-5 (PMC8501694; doi:10.1186/s13023-021-02035-5)
Supplement: Supplementary file 1 — Additional file 1: Table S1. Literature search strategy executed on 23 May 2014. Table S2. Description of experts. [file 13023_2021_2035_MOESM1_ESM.docx]

Supplementary materials

Table S1. Literature search strategy executed on 23 May 2014

| Search | Results | Type |
| --- | --- | --- |
| 1. (systemic mastocytosis).ab | 2051 | Search keywords in abstracts only |
| 2. (signs OR symptoms).ab | 1676951 | Search keywords in abstracts only |
| 3. (aggressive or advanced or indolent or smoldering or AHNMD or “mast cell leukemia” or “mast cell leukaemia” or subtype).af. | 1332583 | Search keywords in all fields |
| 4. 1 AND 2 AND 3 | 254 |  |
| 4. Limit 4 to abstracts | 254 | Limits |
| 5. Limit 4 to English language | 210 | Exclude non-English articles |
| 6. Limit 5 to humans | 189 | Exclude articles describing non-human studies |
| 7. Remove duplicates | 125 | Deduplication |

Databases: Ovid MEDLINE(R) 1946 to Present with Daily Update; Embase 1988 to 2014 Week 20; PyscINFO 1967 to 2014 week 3; Date of search: 23 May 2014

Table S2. Description of experts

| Expert  (systemic mastocytosis subtype[s] discussed) | Specialty | Setting | Years treating systemic mastocytosis patients | Approx. # of AdvSM patients treated per year | Approx. # of ISM patients treated per year |
| --- | --- | --- | --- | --- | --- |
| Expert 01  (AdvSM, ISM) | Allergy and immunology | Academic hospital | 15 | 10–15 | 60–70 |
| Expert 02  (ISM) | Allergy and immunology | Hospital | 23 | “A few” | 200–300 |
| Expert 03  (AdvSM) | Oncology | Cancer center | 13 | 20–50 | 20–30 |
| Expert 04  (AdvSM, ISM) | Myeloproliferative disorders | Academic hospital | 9 | 5–6 | 29–30 |
| Expert 05  (AdvSM, ISM) | Hematology | Academic hospital | 11 | 3–5 | 15 |
